# Validation of the Pearlin Mastery Scale for unpaid caregivers of people living with dementia in Brazil: a methodological study

**DOI:** 10.1590/1516-3180.2024.0446.R1.13062025

**Published:** 2025-08-25

**Authors:** Gustavo Carrijo Barbosa, Diana Quirino Monteiro, Ana Carolina Ottaviani, Camila Rafael Ferreira Campos, Ludmyla Caroline de Souza Alves, Gabriela Martins, Larissa Corrêa, Luana Aparecida da Rocha, Sofia Cristina Iost Pavarini, Elizabeth Joan Barham, Fabiana de Souza Orlandi, Keila Cristianne Trindade da Cruz, Deborah Oliveira, Aline Cristina Martins Gratão

**Affiliations:** I Physiotherapist. Programa de Pós-Graduação em Enfermagem, Universidade Federal de São Carlos (UFSCar), São Carlos (SP), Brazil; II Gerontologist. Programa de Pós-Graduação em Gerontologia, Universidade Católica de Brasília (UCB), Brasília (DF), Brazil.; III Gerontologist. Departamento de Medicina, Universidade Federal de São Carlos (UFSCar), São Carlos (SP), Brazil.; IV Psychologist. Programa de Pós-Graduação em Psicologia, Universidade Federal de São Carlos (UFSCar), São Carlos (SP), Brazil.; V Gerontologist. Programa de Pós-Graduação em Enfermagem, Universidade Federal de São Carlos (UFSCar), São Carlos (SP), Brazil.; VI Gerontologist; PhD student, Programa de Pós-Graduação em Enfermagem, Universidade Federal de São Carlos (UFSCar), São Carlos (SP), Brazil.; VII Gerontologist; PhD student, Programa de Pós-Graduação em Enfermagem, Universidade Federal de São Carlos (UFSCar), São Carlos (SP), Brazil.; VIII Gerontologist. Programa de Pós-Graduação em Enfermagem, Universidade Federal de São Carlos (UFSCar), São Carlos (SP), Brazil.; IX Nurse; Professor, Departamento de Enfermagem; Departamento de Gerontologia, Universidade Federal de São Carlos (UFSCar), São Carlos (SP), Brazil.; X Psychologist; Professor, Departamento de Psicologia, Universidade Federal de São Carlos (UFSCar), São Carlos (SP), Brazil.; XI Nurse; Professor, Departamento de Enfermagem; Departamento de Gerontologia, Universidade Federal de São Carlos (UFSCar), São Carlos (SP), Brazil.; XII Nurse; Professor, Departamento de Enfermagem, Universidade de Brasília (UnB), Brasília (DF), Brazil.; XIII Nurse; Professor and Researcher, Universidad Andrés Bello, Millennium Institute for Care Research (MICARE), Santiago, Chile.; XIV Nurse; Professor; Departamento de Enfermagem; Departamento de Gerontologia, Universidade Federal de São Carlos (UFSCar), São Carlos (SP), Brazil.

**Keywords:** Validation study., Nursing assessment., Caregivers., Coping skills., Dementia., Sense of mastery., Mental health., Validation of instruments., Major neurocognitive disorder., Family caregiver.

## Abstract

**BACKGROUND:**

Sense of mastery has been described in the literature as a psychological resource potentially associated with reduced caregiver burden. However, there are no validated instruments in the Brazilian context that allow the evaluation of the sense of mastery as a possible outcome of interventions aimed at supporting unpaid caregivers of people with dementia.

**OBJECTIVE:**

To validate the construct of the Brazilian version of the Pearlin Mastery Scale among unpaid caregivers of people living with dementia.

**DESIGN AND SETTING:**

This methodological study was conducted with 100 unpaid caregivers of people living with dementia, cared for at home, and who resided in Brazil.

**METHODS:**

Evidence of validity was tested based on internal structure, reliability, and correlations with theoretically related constructs. Sociodemographic data on sense of mastery, burden, depressive symptoms, anxiety, and quality of life were collected.

**RESULTS:**

Difficulties involving burden (80%), depressive symptoms (70%), anxiety (65%), and low quality of life (62%) were reported. Satisfactory measures of adjustment were found in the factor analysis; however, by removing two inversely scored items, these measures improved, resulting in a Cronbach’s alpha of 0.75. Significant correlations were found between sense of mastery and burden scores (ρ = −.56), symptoms of depression (ρ = −.57), anxiety (ρ = −.57), and quality of life (ρ = .64).

**CONCLUSION:**

Evidence of validity was found for the Pearlin Mastery Scale Brazilian version based on the internal structure, reliability, and correlations with theoretically related constructs, indicating that it is a suitable instrument for use in Brazil.

## INTRODUCTION

 A sense of mastery is a psychological coping mechanism that reflects an individual’s self-perception of control over the circumstances that affect their life. Unlike constructs that fall under beliefs and attitudes, such as self-efficacy, mastery is a more global concept and does not apply to specific situations or tasks. People with a high mastery level cope better with chronic stressful experiences, representing a protective factor for mental and physical well-being.^1-4^ Unpaid caregivers of people living with dementia can have positive experiences that represent gains during care-related activities, such as strengthening their sense of mastery. In other words, caregivers perceive that there are important psychological benefits to this experience. In addition to a greater sense of mastery, other positive psychological effects of caregiving already documented in the literature include an increase in optimism, satisfaction, self-esteem, self-efficacy, and resilience.^2 ,4-6^ Some authors suggest that a high level of sense of mastery acts as an important psychological mechanism that can mitigate the effects of the burden generated by care activities.^3,6,7^

 Considerations of the positive effects of care are relatively new. In general, researchers who have evaluated the effectiveness of caregiver support interventions have focused on verifying the reduction in the negative impacts of caregiving, such as burden, depression, and anxiety.^8,9^ Thus, there is little information on the positive effects of the experience of caring for people living with dementia, such as a sense of mastery. These effects also need to be investigated, as they provide a different look at the ways in which interventions can make a difference in the lives of caregivers.^10^ However, instruments to assess the sense of mastery have not yet been adapted for use in Brazil. The Perlin Mastery Scale is an internationally recognized instrument, with multiple studies from various countries providing satisfactory evidence of its validity. It has also been effectively applied in research across diverse contexts and populations.^11-14^ Therefore, the validation of this instrument would allow the evaluation of the sense of mastery in Brazil, as well as the integration of Brazilian information with international literature. 

 The Brazilian Pearlin Mastery Scale (PMS-BR) is a one-dimensional instrument comprising seven items, originally developed by Pearlin and Schooler in the 1970s for a study on stress and coping with a general population sample aged between 18 and 65 years.^1^ Since then, the instrument has been used to assess the sense of mastery in studies that show evidence of the scale for research and practical implementation. In Brazil, translation and cultural adaptation of the PMS were conducted specifically for unpaid caregivers of people living with dementia.^ 3,13- 15^ Given this, it is essential to analyze the evidence of validity for the PMS-BR to determine whether it can effectively evaluate the outcomes of interventions and support programs for caregivers in Brazil. Based on these considerations, this study aimed to assess the construct validity of the PMS-BR through confirmatory factor analysis and to examine its reliability among unpaid caregivers of individuals living with dementia. 

## METHODS

### Study design

 This was a methodological study of construct validation through confirmatory factor analysis of the PMS-BR.^15^ Methodological studies are focused on the analysis and validation of measurement instruments, ensuring that they are appropriate for the population and context in question. This study aimed to assess the structural validity of the PMS-BR and verify its psychometric properties (such as validity and reliability) to ensure its application in a Brazilian context. Data were collected between January 2023 and April 2023. This study is a branch of an umbrella project evaluating the effects of the iSupport-Brazil program on unpaid caregivers of people living with dementia in Brazil.^16^

### Participants

 This study used a convenience sample of 100 unpaid caregivers of people living with dementia from various regions of Brazil. The sample size resulted in a ratio of more than 14 participants per item of the instrument being analyzed, which is a commonly accepted sampling approach, typically ranging from 7 to 10 participants per item, ensuring that the instrument analysis would be reliable.^17^

### Selection criteria

 The participants were people aged ≥ 18 years, who considered themselves to be unpaid carers of people living with dementia, had been doing so for at least six months, residing in any location in Brazil, who cared for a person with a dementia diagnosis (selfreport and Ascertaining Dementia Interview [AD8] score ≥ 2) and had access to a smartphone, computer or tablet with internet. In addition, only participants with scores above the minimum values established for at least two of the following three measures were included in the study: ≥ 4 points in the assessment of overall burden perception, or ≥ 3 points in the assessment of anxiety symptoms, or ≥ 3 points in the assessment of depression symptoms. Caregivers of people living in long-term care institutions were excluded. These criteria were defined based on the iSupport-Brazil umbrella project, of which this study is a part. 

### Recruitment and data collection

 Participants were recruited through posters, flyers, social media (Instagram and Facebook), and partnerships with associations affiliated with the Brazilian Federation of Alzheimer’s (Febraz), such as the Brazilian Alzheimer’s Association (ABRAz), and researchers from higher education institutions and public health services across Brazil who promoted the study on their websites, social media, and bulletin boards within their institutions. Individuals interested in participating in the study completed an online pre-registration (via Google Forms), providing information such as full name, email address, and WhatsApp details. The researchers then contacted the participants and sent them a link to an online form (via Google Forms) regarding the study’s eligibility criteria. Participants who met the criteria received another link to a different form (via Google Forms) for data collection. This form included information about the study and items of the Informed Consent Form. Once consent was obtained, the participants answered the study questions. The entire recruitment and data collection process was conducted online. 

## STUDY VARIABLES

 Sociodemographic and care characteristics: sex, age, marital status, skin color, region of residence, education, relationship with the person in need of care, time working in the position in days/week and hours/day, and type of dementia. 

 PMS-BR: The PMS-BR is a one-dimensional instrument of seven items scored on Likert scales from "1 = strongly agree" to "4 = strongly disagree", using reverse coding for items 4 and 6 of the scale, which are worded positively. The total score varies between seven and 28 points; the higher the score, the greater the sense of mastery. 

 Perceived burden: For inclusion in the study, a single item was answered on general burden perceptions (on a 10-point Likert scale): "On a scale from 1 to 10, where 1 means ’not burdened’ and 10 means ’extremely burdened’, how burdened do you feel?". The participants answered the Zarit Burden Interview (ZBI) questionnaire to assess the subjective burden related to caring for another person using 22 items scored on a Likert scale from 0 to 4 points. The total score ranges from zero to 88, with higher scores indicating a greater perception of burden.^18,19^

 Depression and anxiety symptoms: The Hospital Anxiety and Depression Scale (HADS) was used,^20^ and adapted with validity evidence for use in Brazil.^21^ The scale has satisfactory performance for assessing anxiety and depression among the general population through 14 items (seven specific questions for each construct) scored on a Likert scale from 0 to 3 points.^22^ Results above eight points indicate a possible/probable manifestation of symptoms of depression and anxiety. 

 Quality of life: The Quality of Life Alzheimer’s Disease (QoL-AD) scale,^23^ adapted and with evidence of validity for use in Brazil,^24^ was used to assess the quality of life of unpaid caregivers who assisted people living with Alzheimer’s disease. The instrument consists of 13 items rated on a four-point scale, with a score of 1 assigned to "bad" and a score of 4 to "excellent." The total score ranged from 13 to 52, with higher scores indicating better quality of life.^23^

## DATA ANALYSIS

 Confirmatory factor analysis (CFA) was carried out using MPLUS software version 6.12.0, using the mean and varianceadjusted weighted least squares (WLSMV) method, recommended for models with categorical indicators, and considering the existence of a single factor. The fit measures included the chisquared test (χ^2^), which compared the sample correlation matrix with the correlation matrix estimated under the model (in this case, lower values indicated a good fit); Bentler’s Comparative Fit Index (CFI); the Tucker–Lewis Index (TLI); the Root Mean Square Error of Approximation (RMSEA); and the Weighted Root Mean Square Residual (WRMR). CFI and TLI values (≥ 0.95), RMSEA (< 0.07), and WRMR (< 1) values were adopted as model fit criteria.^25-29^

 The other analyses were carried out using SAS, version 9.4, Cronbach’s alpha was calculated to check the internal consistency of the instruments (satisfactory ≥ 0.7);^30^ Spearman’s coefficient to check the discriminant construct validity through the correlation between the PMS-BR scores and those of the ZBI and HADS; and the convergent analysis between the PMS-BR scores and the QoL-AD scores (considering that a correlation < 0.3 = weak; between 0.3 and 0.5 = moderate; > 0.5 = strong).^31^ A 95% confidence interval was adopted, with statistical significance set at P ≤ 0.05. 

## ETHICAL ASPECTS

 This study was conducted in accordance with the guidelines of Resolutions No. 466/2012 and No. 510/2016 of the Brazilian National Health Council. This study was approved by the Human Research Ethics Committee of the Universidade Federal de São Carlos (Protocol No. 5.332.333; CAAE: 88157118.0.1001.5504). 

## RESULTS

 Of the 100 caregivers who participated, 95% were female, with a mean age of 53.4 years (standard deviation: 9.32 years). Most of them were married (56%), self-declared as white (56%), and lived in the Southeast (65%). Around 90% reported ≥ 12 years of schooling, more than 85% reported being children of the person living with dementia, 40% had been caring for them for between 1 and 3 years, 67% were involved 6–7 days a week and 42% reported caring for 13 or more hours a day. More than 72% of participants reported that they cared for people with Alzheimer’s disease. **Table 1** shows the descriptive statistics of the scores obtained for the measures of burden, depression, anxiety, and perceptions of quality of life. 

**Table 1:** Descriptive analysis of the measures of burden, depression, anxiety, and the participants’ perceptions of quality of life (N^*^ = 100). Brazil, 2023

**Variables**		**n^†^ (%) / M^‡^ (SD^§^ )**
**ZBI^||^**		62.85 (12.6)
	No burden (≤ 20)	5 (5%)
	Light burden (21–40)	38 (38%)
	Moderate to severe burden (41–60)	42 (42%)
	Intense burden (≥ 61)	15 (15%)
**HADS^¶^– Overall score**		18.72 (7.74)
	**HADS^¶^– Depression**	9.48 (3.77)
	Possible/probable depression (≥ 8)	70 (70%)
	No symptoms (≤ 7)	30 (30%)
**HADS^¶^– Anxiety**		9.24 (4.31)
	**HADS^¶^– Depression**	9.48 (3.77)
	Possible/probable depression (≥ 8)	65 (65%)
	No symptoms (≤ 7)	35 (35%)
	QoL-AD ^**^– Overall score	30.54 (7.5)
	Higher levels (≥ 32)	38 (38%)
	Lower levels (< 32)	62 (62%)

* N = total participants

† n = sample size

‡ M = mean

§ SD = standard deviation

|| ZBI = Zarit Burden Interview

¶ HADS = Hospital Anxiety and Depression Scale

** QoL-AD = Quality of Life-Alzheimer Disease

 Initially, although the adjustment measures were satisfactory in the CFA, the standardized factor loading estimate, which indicates the correlation of the variable with the instrument’s single factor, showed lower factor loading values for items 4 and 6 of the scale (≤ 0.52), which are those scored inversely, in addition to item 6 not showing a significant correlation (**Table 2**). 

**Table 2:** Standardized factor loading estimates for each item. Brazil, 2023

	**Estimate**	**Standard error**	P value^*^
1. "I can’t solve some of the problems I have"	0.65	0.061	< 0.001
2. "Sometimes I feel like I’m being forced to do things in life"	0.65	0.069	< 0.001
3. "I have little control over the things that happen to me"	0.69	0.072	< 0.001
4. "I can do anything when I put my mind to it"	0.52	0.076	< 0.001
5. "I often feel incapable of dealing with life’s problems"	0.8	0.054	< 0.001
6. "How I deal with what happens to me in the future will depend mainly on me"	0.19	0.098	0.055
7. "For many important things in my life, there is little I can do to change them"	0.59	0.077	< 0.001

* P value = significance level.

 On identifying this result, a new confirmatory factor analysis model without the inclusion of items 4 and 6 was tested and showed more satisfactory adjustment measures for all the indices, with a significant reduction in the RMSEA and chisquared values, as well as an increase in the CFI and TLI values (**Table 3**). 

**Table 3:** Results of the adjustment measures used for the PMS-BR AFC.^* †^ Brazil, 2023

**Adjustment index**	**Model – version 7 items**	**Model – version 5 items**
RMSEA^‡^	0.071 (90% CI: 0–0.13)	0.047 (90% CI^§^: 0–0.153)
CFI^||^	0.976	0.996
TLI^¶^	0.964	0.991
Chi-squared	21.146 (14)	6.104 (5)
WRMR^**^	0.579	0.372

* CFA = Confirmatory Factor Analysis

† PMS-BR = Brazilian Pearlin Mastery Scale

‡ RMSEA = Root Mean Square Error of Approximation

§ CI = Confidence Interval

|| Bentler’s Comparative Fit Index

¶ TLI = Tucker–Lewis Index

** WRMR = Weighted Root Mean Square Residual

 By reducing the scale to five items, the internal consistency was increased from α = 0.73 to α = 0.75, reinforcing the more satisfactory validity evidence for the PMS-BR reduced version (**Table 4**). 

**Table 4:** Analysis of internal consistency between PMS-BR^*^ items. Brazil, 2023

**Item**	**Correlation between total score and item**	**Value of α if the item is excluded**
1	0.508	0.687
2	0.474	0.693
3	0.525	0.68
4	0.443	0.7
5	0.544	0.677
6	0.161	0.764
7	0.485	0.69

* PMS-BR = Brazilian Pearlin Mastery Scale.

 There was a significant positive correlation of strong magnitude between PMS-BR scores and QoL-AD and a significant negative correlation of strong magnitude between the reduced version PMS-BR scores and ZBI, HADS—Depression, and HADS—Anxiety, establishing evidence for criterion validity based on correlations with theoretically related constructs using the PMS-BR reduced version in Brazil (**Table 5**). 

**Table 5:** Analysis of criterion validity evidence. Brazil, 2023

		ZBI^*^	**HADS ^†^– Depression**	**HADS ^†^– Anxiety**	**QoL-AD^‡^**
PMS-BR	ρ^§^	−0.56	−0.57	−0.57	0.64
	p^||^	< 0.01^¶^	< 0.01^¶^	< 0.01^¶^	< 0.01^¶^
	N^**^	100	100	100	100

* ZBI = Zarit Burden Interview

† HADS = Hospital Anxiety and Depression Scale

‡ QoL-AD = Quality of Life-Alzheimer Disease

§ ρ = Spearman’s correlation coefficient

|| P value = Significance level

¶ statistically significant value

** N = Total participants

## DISCUSSION

 In this study, evidence confirmed the expected unidimensional factor structure, significant correlations with related constructs, and reliability of the PMS-BR seven-item version. However, items 4 and 6 were excluded after finding evidence of a weak relationship between their scores and the factor score for this instrument. After excluding these items, the adjustment measures were more robust for the reduced version of the scale (five items), corroborating other studies,^3,12-14^ which contributes to filling a gap in the lack of instruments to assess the important positive aspects involved in caring activities in Brazil. 

 Despite clear evidence of problems in the interpretation of items 4 and 6, the fit indices for the PMS-BR, the instrument’s reliability, and the correlations with theoretically related constructs were satisfactory. However, it will be necessary to modify the wording of these items and conduct a new validity evidence assessment to determine whether the problem can be remedied. In general, low-magnitude correlations are found when there are different interpretations of the meaning of an item. 

 In relation to the caregivers’ profile, there was a large predominance of female participants in the sample (95%), with a mean age of 53 years, most of whom were white and married, which corroborates the National Report on Dementia in Brazil, which analyzed the context of Brazilian caregivers of people living with dementia and their main needs.^32^ In addition, most of the participants had a high education level and worked as caregivers 6 to 7 days a week, for 13 or more hours a day. Despite changes in gender and cultural patterns, such as a reduction in fertility rates, an increase in education levels among women in the last three decades, and their entry into the labor market, the social responsibility that women assume for care activities is still notable.^33^

 Analysis of the factor loadings for the PMS-BR items found a very low value for items 4 and 6 of the scale (≤ 0.52), corroborating the study carried out to develop the original version of the instrument, in which the authors carried out exploratory factor analysis, in which they observed reduced factor loadings for the items with inverse scores (≤ 0.47), but did not carry out reliability analysis.^1^ In our analysis, the PMS-BR showed satisfactory internal consistency results through Cronbach’s alpha, which showed a value of 0.73 for the full version and 0.75 for the five-item version (without those scored inversely). 

 A study of 392 family caregivers of dependent older adults in Singapore corroborated our findings with satisfactory reliability (0.8), which reflected a lower consistency for the two positively worded items. Without the two items, the analysis of the reduced version showed an acceptable model fit and obtained higher reliability compared to the seven-item version (0.82).^3^ In the study of the instrument’s Canadian version^14^ the reliability analysis of the scale applied to 1,377 older adults showed an alpha of 0.51 to 0.54 for the seven-item version, rising to 0.72 to 0.76 in the analysis of the fiveitem version. The same is true of the psychometric analysis of the Japanese version of the scale, conducted with 2,067 residents aged between 25 and 74 years, which showed better internal consistency for the version without inversely scored items, from 0.69 to 0.77.^12^

 Although the difference in reliability between the seven-item and five-item scales was not as pronounced in the present sample, further investigation of the reduced version (five items) confirmed that this version provides a better fit to the model than the seven-item scale, as observed in previous studies.^3,12-14^ When reviewing the instrument’s structure, one possible reason for the poor performance observed in the withdrawn items could be the effects of their inverted wording, which makes it difficult to measure the construct they were intended to measure.^3,11^ The study of translation and cultural adaptation of the instrument to its Brazilian version considered all items of the original scale. Through factor analysis, this study showed that the removal of items with inverted scores demonstrated greater reliability and a better model fit. Future studies could consider changing the wording of positively worded items to understand whether their performance is related to qualitative aspects or is purely an effect of the method. 

 Correlation analysis between sense of mastery and perceived burden, depressive symptoms, anxiety, and quality of life indicated that the PMS-BR-reduced version has validity based on relationships with related constructs. Given that sense of mastery is an important psychological mechanism that mediates burden and is an important protector of mental well-being,^3,12^ these results indicate that the PMS-BR has criterion validity. Therefore, having a high level of mastery is important for caregivers of people living with dementia, as this context implies vulnerability to anxiety and depression, influencing their burden perception.^4,12,34,35^

 The negative correlation between the sense of mastery and the negative impacts of caring can be attributed to the psychological protection provided by higher mastery levels compared to those with lower mastery levels, which also results in less manifestation of psychological symptoms such as depression and anxiety.^3^ Similarly, the positive correlation between the sense of mastery and the caregivers’ quality of life reinforces the protective role of this coping mechanism. Other studies corroborate this finding and point to a relationship between mastery and other positive care aspects, such as positive self-perception of health, self-efficacy, and life satisfaction, suggesting a sense of mastery as a mediating phenomenon between perceived burden and psychological symptoms.^3,12,36,37^

 A limitation of this study concerns the selection of participants, which was restricted by inclusion criteria based on specific scores on certain measures. Additionally, since this study is part of a larger project, the sample may have been further limited and might not fully represent the diverse experiences of caregivers of people with dementia in Brazil. Another relevant limitation is the linguistic nuances of Brazilian Portuguese spoken in different regions of Brazil. The PMS-BR version may have difficulty in capturing linguistic and cultural variations in mastery perceptions among caregivers, especially considering the vast cultural and regional contexts of Brazil. The different forms of expression and interpretation of terms related to mastery and burden may have influenced the responses in a non-uniform manner. Therefore, future research should consider the regional particularities of Brazilian Portuguese, addressing both the translation and cultural validation of assessment instruments. 

## CONCLUSION

 The assessment of the sense of mastery through the PMS-BR in unpaid caregivers of people living with dementia in Brazil provides a valuable contribution to the theoretical understanding of this construct in caregiving contexts. The construct validation and reliability of the PMS-BR were successful, demonstrating its high reliability for evaluating the effectiveness of interventions aimed at supporting caregivers. 

 These findings reinforce the relevance of mastery as a psychological coping mechanism, mitigating the effects of caregiver burden and psychological symptoms. Future research should continue to explore and strengthen the theoretical framework surrounding the sense of mastery in this population, with additional studies validating the instrument and examining its potential impact on caregiving strategies and outcomes. 
